# Mental Effort When Playing, Listening, and Imagining Music in One Pianist’s Eyes and Brain

**DOI:** 10.3389/fnhum.2020.576888

**Published:** 2020-10-15

**Authors:** Tor Endestad, Rolf Inge Godøy, Markus Handal Sneve, Thomas Hagen, Agata Bochynska, Bruno Laeng

**Affiliations:** ^1^Department of Psychology, University of Oslo, Oslo, Norway; ^2^RITMO Centre for Interdisciplinary Studies in Rhythm, Time and Motion, University of Oslo, Oslo, Norway; ^3^Helgelandssykehuset, Mosjøen, Norway; ^4^Department of Psychology, New York University, New York, NY, United States

**Keywords:** imagery, music, audiation, audio-visual integration, pupillometry, fMRI

## Abstract

We investigated “musical effort” with an internationally renowned, classical, pianist while playing, listening, and imagining music. We used pupillometry as an objective measure of mental effort and fMRI as an exploratory method of effort with the same musical pieces. We also compared a group of non-professional pianists and non-musicians by the use of pupillometry and a small group of non-musicians with fMRI. This combined approach of psychophysiology and neuroimaging revealed the cognitive work during different musical activities. We found that pupil diameters were largest when “playing” (regardless of whether there was sound produced or not) compared to conditions with no movement (i.e., “listening” and “imagery”). We found positive correlations between pupil diameters of the professional pianist during different conditions with the same piano piece (i.e., normal playing, silenced playing, listen, imagining), which might indicate similar degrees of load on cognitive resources as well as an intimate link between the motor imagery of sound-producing body motions and gestures. We also confirmed that musical imagery had a strong commonality with music listening in both pianists and musically naïve individuals. Neuroimaging provided evidence for a relationship between noradrenergic (NE) activity and mental workload or attentional intensity within the domain of music cognition. We found effort related activity in the superior part of the locus coeruleus (LC) and, similarly to the pupil, the listening and imagery engaged less the LC–NE network than the motor condition. The pianists attended more intensively to the most difficult piece than the non-musicians since they showed larger pupils for the most difficult piece. Non-musicians were the most engaged by the music listening task, suggesting that the amount of attention allocated for the same task may follow a hierarchy of expertise demanding less attentional effort in expert or performers than in novices. In the professional pianist, we found only weak evidence for a commonality between subjective effort (as rated measure-by-measure) and the objective effort gauged with pupil diameter during listening. We suggest that psychophysiological methods like pupillometry can index mental effort in a manner that is not available to subjective awareness or introspection.

## Introduction

### Musical Effort

The piano is not an easy instrument to master. It requires a lifetime of extended practice and early (possibly during childhood) onset of training to achieve a high level of performance. Well-known musical pieces in either classical music or jazz are technically particularly challenging, and they constitute a paradigmatic example of intense practice of cognitive executive functions (Montero, 2016). There is the need of dividing attention over the control of complex, synchronized, sequences of finger positions and movements (Mikumo, 1994) and paying attention to the obtained auditory stimuli. Such a fine and precise motor control of hands and fingers require cognitive and motor control, guiding constant adjustments of bodily actions for both the execution and preparation of the following movements during the different passages of a musical piece, also depending on the level of cognitive and motoric demands in performing the movements. Hence, we assume that playing the piano—or any musical instrument professionally—requires mental resources or “mental effort,” as Kahneman (1973) labeled it originally. Such a type of neurocognitive effort is distinguishable from physical effort, though it has clear analogies with it.

Surprisingly, research on “musical effort” or the broad process of allocation of attentional resources during music performance or listening (Keller, 2001; Shenhav et al., 2017) has been so far an understudied aspect of music cognition. In cognitive psychology, pupillometry or the measurement of pupil dilations during a task has been considered the best psychophysiological measure (Kahneman et al., 1969) of the “intensive aspect of attention” or cognitive workload (e.g., Hess and Polt, 1964; Beatty, 1982; Just and Carpenter, 1993). Changes in pupil diameter are not simply evoked by changes in light stimulating the eye, and changes related to mental processing, albeit tiny compared to those provoked by light, are separable. Most importantly, these pupillary changes provide a reliable, “honest” (i.e., difficult to affect voluntarily; see Laeng and Sulutvedt, 2014), and valid measure of the overall aggregate of resource demand and capacity utilization by the brain (Just et al., 2003). A wide variety of pupillometry studies confirm a tight relationship between pupillary dilation and the allocation of attention and load on cognitive resources (for reviews see Laeng et al., 2012; Laeng and Alnæs, 2019). Recently, neurophysiological studies with monkeys (e.g., Joshi et al., 2016) and neuroimaging in humans have indicated the involvement of the noradrenergic (NE) brainstem structure called the locus coeruleus (LC) in the control of pupil size during cognitive work (e.g., Alnæs et al., 2014; Murphy et al., 2014; Mäki-Marttunen et al., 2019). Both pupil diameter and the LC have been known as a physiological measure and structure involved in both cognitive and affective “arousal” (e.g., Nunnally et al., 1967; Libby et al., 1973; Nassar et al., 2012; Sara and Bouret, 2012).

Notably, even just music listening triggers high arousal (e.g., Gingras et al., 2015; Laeng et al., 2016; Weiss et al., 2016; Bowling et al., 2019). To our knowledge, no studies have applied eye pupillometry to the study of “musical effort” during the performance. Instead, there have been a few pupillometry studies on mental effort during the perceptual processing of auditory stimuli like speech (e.g., Zekveld et al., 2014) and music (e.g., Kang and Wheatley, 2015; O’Shea and Moran, 2016; Liao et al., 2018). Moreover, it remains unclear whether the cognitive mechanisms engaged during music listening are equally engaged during musical auditory images (e.g., when a musician “plays in her head” a musical piece) than with music perception or when listening to the same musical piece played on a real instrument. Research within cognitive psychology had mainly focused on visual imagery (e.g., Kosslyn, 1980, 1994), which has overall confirmed a strong overlap of the cognitive mechanisms engaged by both imagery and perception.

### Musical Imagery

Musical imagery consists of actively evoking and maintaining sound “images” of music in our minds, like mental imagery in other specific modalities (Godøy and Jørgensen, 2001). However, musical imagery would seem to be a process at the very heart of not only music-making but also listening, as an ongoing anticipatory activity (e.g., Janata, 2001; Leaver et al., 2009; Gracyk, 2019). An interest in the topic has been rekindled within the last 20 years by several neuroimaging studies investigating the effects of music perception and imagery in the brains of both musicians and non-musicians (e.g., Zatorre, 1999; Lotze et al., 2003; Herholz et al., 2008). Recently, most references to musical imagery have focused on “involuntary musical imagery” (IMI, sometimes called “earworms,” “sticky music,” “catchy tunes,” or simply “hooks”; e.g., Sacks, 2007; Farrugia et al., 2015; Williams, 2015; Moseley et al., 2018). However, in this article, we will focus on the “volitional” or active type of imagery of musical sounds and report a psychophysiological plus neuroimaging study on imagery, listening, and performance by a professional, internationally renowned, pianist.

#### Musical Imagery as a Multimodal Experience

Importantly, the phenomenology of music-making and listening is not just related to sounds or finger movements but it is a multisensory or multimodal experience (Vines et al., 2006; Godøy, 2010b; Zimmerman and Lahav, 2012; Fine et al., 2015). The musical imagery of musicians seems particularly embedded in complex action plans, co-articulated executive programs, and predictions of the effects of actions both within the body and in the environment (e.g., Reybrouck, 2001; Wolpert and Flanagan, 2001). Musicians seem capable of constructing “sonic images” when playing, based on timbral and timing features (Wöllner and Williamon, 2007), allowing them to anticipate sonic actions and even perform without auditory feedback (e.g., with sound is switched off). However, also non-musicians appear to have an intuitive and coarsely correct understanding of the visual features linked to playing a particular musical instrument and of its related specific gestures or “gestural affordances” (Godøy and Leman, 2010; Godøy, 2010a); as also testified by the amusing ability of non-musicians in “playing” virtual instruments (e.g., “air-guitar”; see Godøy, 2001; Godøy et al., 2006). Just the sight of the musicians’ gestures (with no sound) can influence our understanding and evaluation of music (e.g., Platz and Kopiez, 2012; Tsay, 2013) and it also triggers activity in the auditory cortex of the observers’ brain (Haslinger et al., 2005), probably indicating that the sight of music-making evokes in turn spontaneous musical (auditory) imagery.

Although we can in principle study in isolation each modality-specific type, given the underlying modularity of the sensory and motor system, there is likely to be a great interconnectedness between processes and structures of the brain for musical imagery. The current picture is that the auditory type of imagery is active together with the motor imagery of the sound-producing body motions. Moreover, the motor imagery seems imbued with somesthetic images, characteristic of the kinesthetic feedback that such bodily motions would typically produce (Betts, 1909; Hubbard and Stoeckig, 1988; Reisberg, 1992; Hodges, 2009; Hubbard, 2010, 2013, 2017, 2018; Vlek et al., 2011; Vuvan and Schmuckler, 2011). Indeed, perception, in general, is “active” and makes use of the motor system to achieve perceptual categorization or, at least, to facilitate it (e.g., Liberman et al., 1967; Liberman and Mattingly, 1985; Smith et al., 1995; Gallese and Metzinger, 2003; Gallese and Lakoff, 2005; Galantucci et al., 2006; Glenberg and Gallese, 2012). Embodied cognition accounts give prominence to action and behavior for perceptual processes of all kinds (e.g., Varela et al., 1991; Gallagher, 2005), including especially music (e.g., Cox, 2016). These accounts have influenced music psychology (Leman, 2008; Peñalba, 2011; Schiavio et al., 2014; Korsakova-Kreyn, 2018; Bailes, 2019). We believe that studying volitional musical imagery is important because it could be the gateway to the more systematic exploitation of musical imagery in practical tasks such as composition, improvisation, and performance (Christensen, 2019).

#### Musical “Audiation”

A remarkable phenomenon of musical imagery is the so-called notational “audiation” often reported by professional musicians; that is, the simple act of reading a musical score evokes auditory imagery of the music in reading’s real-time (e.g., Schürmann et al., 2002; Battisti, 2007; Brodsky et al., 2003, 2008). Remarkably, the electrophysiological activity from the brain of trained musicians during note reading or the actual perception of notes is undistinguishable (Simoens and Tervaniemi, 2013). The ability to “hear” with the “mind”s ear’ or “thinking in sounds” (Combarieu, 1907) and “replay” virtual music with the “inner voice,” could assist the making of creative compositions. A well-known case of the power of musical imagery in creating music is Ludwig van Beethoven who composed many of is most praised compositions (e.g., the last piano sonatas and string quartets, the Missa Solemnis, and the Ninth Symphony) while he was practically deaf due to an inner ear problem. Presumably, he was perfectly able to compose music because he could “hear the music in his head” (Jourdain, 1997; Zatorre and Halpern, 2005) despite his spared auditory cortex was unable to be stimulated by the actual sounds of musical instruments.

Hearing music in the head appears to be a ubiquitous experience (Cotter, 2019) but the role that mental control or effort plays in these experiences has not been addressed in current research in any thorough manner. That is, both professional musicians and music writers, as well as musically naïve individuals, have the power to start, stop, shape, and maintain in their head musical images unfolding in time (e.g., Zatorre, 1999; Janata, 2001; Cotter, 2019). However, all these mental “actions” are a form of cognitive work and require focused attention; they are likely to draw on mental resources and engage brain systems the control cognitive arousal (Alnæs et al., 2014). Indeed, musical “mental practice” (Coffman, 1990) is well known as an effective tool for enhancing memorization of music and refining performance (e.g., Driskell et al., 1994; Halpern et al., 2004; Highben and Palmer, 2004; Holmes, 2005; Lotze and Halsband, 2006; Cahn, 2008; Gregg et al., 2008; Keller, 2012; Halpern and Overy, 2019). Deliberately imagining music, both the sound-producing actions (e.g., finger movements in pianists) and the resulting musical sounds (Davidson-Kelly et al., 2015), seems almost as common as actually listening to music (respectively 32 and 44% of times when querying musicians; Bailes, 2006, 2015). According to Fine et al. (2015), about 70% of the classical music performers they surveyed state that they use regularly mental practice, and among these about 90% also experience musical imagery in the form of “audiation” (i.e., “hearing music in the head” while reading a score away from the instrument; Bishop et al., 2013). A well-known case of extensive use of the mental practice is that of the classical pianist Glenn Gould who mentally rehearsed a performance, without touching the piano for prolonged periods, leaving the actual testing of the “mechanics” of the finger movements on the piano to just the last period of preparation (Mesaros, 2008). In a seminal study, Repp (1999) compared the timing profiles of six pianists during a live performance and the “imagined performance” and found that the temporal fluctuations occurred in a very similar manner (i.e., they were positively correlated). Other experiments in “mental chronometry” of imagined music have yielded similar results (Wöllner and Williamon, 2007; Clark and Williamon, 2011; Clark et al., 2012).

### The Present Study

Because a piano piece can present cognitive challenges that rapidly vary during the music’s stream, we expect that changes in pupillary diameter in the eyes of the pianist mirror the level of required effort as the music unfolds. We expect that the complete trace of pupillary changes will provide a continuous physiological measure of changes in control processes (executive and attentional) occurring in the brain of a high-level professional pianist as she focusses on the piece over time. We also expect that, to some extent, independent listeners would react to the perceived effort inherent in the music, likely more in musicians than in non-musicians. Hence, we also monitored the eye pupil in a “control” group of pianists and non-musicians, while they listened to the same piano renditions of the musical pieces by our professional pianist.

Importantly, the pupillometry method is currently considered not only a reliable gauge of cognitive workload but also as a window into the activity of the brain’s NE arousal system and the involvement of brainstem structures like the LC, involved in the NE control of pupil size (e.g., in other mammals; Joshi et al., 2016). Very few human neuroimaging studies have explored the role of the brainstem’s NE structures and pupillary activity (Alnæs et al., 2014; Murphy et al., 2014; Mäki-Marttunen et al., 2019) and none have related the role of these brainstem’s NE structures to music listening performance, and imagery. Hence, we further explored music listening, playing, and imagery, in the same professional pianist, during these tasks with functional MRI, seeking converging evidence to the engagement of mental effort by brainstem structures. Since musical imagery is a quintessential multisensory experience (auditory, kinesthetic, and visuospatial at least), playing music in one’s head or imagery should reveal the strongest relationship with actual piano playing, since all of the components of music-making that takes place in the mind (brain) would be re-instated, despite the lack of behavioral enactment. Importantly, we expect to reveal converging evidence between the findings obtained with pupillometry (Experiment 1) and those obtained with neuroimaging (Experiment 2). Specifically, since we posit that the pupil index effort-related cognitive arousal, we expect that pupillary changes across conditions should also appear as changes in activity in the LC or the NE center of the brainstem (Alnæs et al., 2014). Moreover, processes that are similar in terms of pupillary activity and highly correlated should also appear similar in terms of cortical activity.

In sum, we hope to offer initial answers about some fundamental questions in music cognition: (a) Can we measure musical effort through the eye pupil similarly to reading out the cognitive workload and arousal in other domains? (b) Does musical imagery engage sensory and motor areas of the brain concerning the mental effort required by the complexity of the structure and execution of the imagined music? (c) Is musical imagery more similar to music listening in non-musicians in terms of neural networks than in musicians where it engages more motor aspects (i.e., it is multimodal)? (d) Can we in general deduce the degree of functional similarity between playing, imagining, and listening by comparing the activity in the NE system (as indexed by the pupil) and/or the overlap between sensory and motor neural networks in the whole brain? and (e) Specifically, can we reveal “audiation” in an expert musician by the similarity in which the pupil changes during musical listening and imagery?

#### Pupillometry of Playing, Listening, and Imagining Music

Pupillometry measures “objectively” mental effort, but an experienced musician may be able to estimate “subjectively” how effortful a moment can be during the piano execution. Hence, we introduce a distinction between “subjective effort” and “objective effort”. With the former, we mean what a participant judges to be the processing load based on her private experience, even when estimated *via* ordinal scales (e.g., the NASA-TLX; e.g., Chaffin, 2009). Instead, physiologically driven changes in pupil diameter are an objective measure of mental effort (Kahneman, 1973) since—differently from verbal reports and ratings—they are not under volitional control (Loewenfeld, 1999; Laeng and Sulutvedt, 2014). Given our assumption that musical imagery is intimately linked with the motor imagery of sound-producing body motions, one straightforward expectation is that there will be a close affinity in load on cognitive resources between standard playing and silent playing. Both involve the planning and execution of complex coordinated body motions and both result in actual movement, muscular deployment, and related metabolic expenditure. This would also be consistent with much evidence from pupillometry research indicating that a simple, single, keypress (used in many paradigms to indicate the detection of a target stimulus or a discriminatory choice) results in measurable dilation of the eye pupil (e.g., Simpson, 1969; Simpson and Climan, 1969). However, overt behaviors and muscles’ contractions are not necessary for pupillary dilations to occur and there is overwhelming evidence in the literature that dilations can index the presence and degree of internal (cognitive or affective) processes without the need for overt responses (e.g., Kahneman and Beatty, 1966; Laeng et al., 2016).

In the present study, we opted for correlational analyses of the measure-by-measure changes in pupil diameter across conditions within each musical piece. We expect that the pupil sizes will co-vary positively across the different phases of a musical piece as a support to the hypothesis of functional overlap. Similarly, there should be a chronometric correspondence between the time required to perform a musical piece and that required for its execution, but this does not imply that the absolute times cannot differ, even when there is strong functional overlap. Based on the theory and findings of the original “mental scanning” experiments (e.g., Kosslyn et al., 1978; Borst and Kosslyn, 2008), the time to scan increasing distances increases at comparable rates in perception and visual imagery. However, when generating mental images from long-term memory, participants could scan more slowly in the mental image condition. Hence, we expect the time course of imaging to be slower for musical imagery than in listening or playing. This seems also to be the case for the time taken to imagine an action and its actual execution in athletes (e.g., Reed, 2002).

Previous studies with the pupillometry method have shown its ability to measure the moment-by-moment changes in cognitive and affective arousal during music listening (e.g., Laeng et al., 2016; Bowling et al., 2019). To our knowledge, there are no previous pupillometry studies during a musical performance. The present use of pupillometry as a gauge of mental work in music seems novel and potentially fruitful.

## Experiment 1A: Pupillometry

If similar processes and mechanisms underlie perception and imagery, executed and imagined movements, then we reason that this functional overlap should be reflected in the level of allocation of cognitive resources and, in turn, in changes in the pupil diameter. Moreover, the degree of effort required during a musical piece may be visible not only in the eyes of a performer but also in those of the listeners. The experience could modulate this response, being stronger in musicians with experience with the same instrument than in the non-musicians. Hence, in this study, we explore how the pianist’s eye pupil can index effectively the level of cognitive resources required in a performance by using differentially challenging musical piano pieces and annotating the momentary difficulty, at different points in time during a musical performance. Also, we monitor the pupils of listeners, either pianists or non-musicians, while listening to the pianistic performances of the professional musician.

We expect positive correlations between pupil diameter changes of the professional pianist during different “executions” (i.e., normal, silenced, listened, and imagined) of the same piano piece, which would indicate the presence of similar task demands and load on resources and the engagement of similar cognitive and execution mechanisms. Conversely, a lack of correlation between pupil diameter changes in the different “executions” of the same piano piece (i.e., normal, silenced, listened, and imagined) might indicate the absence of common mechanisms but the strength of the correlations should give a hint to the degree of functional overlap or equivalence.

### Method

We use the pupillometry method based on infrared eye-tracking, which allows the precise tracking of the size of the pupil in both eyes simultaneously in a non-invasive manner and with no restrictions on eye movements, at a sampling rate equal or superior to standard film or television (e.g., PAL is 50 Hz).

#### Participants

This first experiment involved the single case of a professional pianist (PP hereafter) who is a music teacher in Oslo, Norway, and an internationally renowned performer (e.g., at Carnegie Hall), with expertise in 19th century piano music and technique. PP is a 41 years old female and she has played the piano since the age of six. Based on her responses to The Goldsmiths Musical Sophistication Index (v1.0), she has a top score of 36 in musical training and a General Sophistication score = 88. PP plays five or more hours per day on her primary instrument (piano), but she can play four other additional instruments. She is highly active in perfecting her technique and has lectured and published on piano techniques from previous centuries. Hence, PP qualifies as exceptional and a true expert in music performance according to the criteria of Montero (2016) and Høffding (2019) and most definitions of expertise regardless of the domain (e.g., for elite sports; Swann et al., 2015). The Department of Psychology’s IRB at the University of Oslo approved the present study (Reference number: 3568281) and PP as well as the other participants (in Experiments 1B and 2B) received a consent form before testing and were treated according to the Declaration of Helsinki.

#### Musical Stimuli

PP provided us with three piano pieces of different technical and interpretive difficulty: an easy piece, a middle-level one, and an advanced one. She chose two compositions of Edvard Grieg: Wächterlied, from Lyrical Pieces, Op.12 No.3, and Holberg Suite, Op. 40, Praeludium (Allegro vivace); these are considered as an easy and a difficult piano piece, respectively. The medium difficulty composition was Robert Schumann’s Träumerei (from Kinderszenen or “Scenes from Childhood,” Op. 15). Each piano piece was performed twice on an electronic keyboard set to “grand piano,” once normally, “standard playing” (hereafter) and once with no sound (by turning off the audio). Also, PP listened to her normal renditions of each piece (as registered in a MIDI file) as well as in a condition where there was neither auditory nor kinesthetic feedback from finger movements, i.e., the “imagery” (hereafter) condition. In all the four conditions, we showed the first two pages of the musical score on the computer screen of the eye-tracking device.

#### Apparatus

We used a Yamaha electronic or digital piano (P-140) set to “grand piano.” The P-140 keyboard has 88 graded-hammer keys with sounds based on Yamaha’s AWM sampling technology, with the convincing similarity of sound to a real acoustic instrument. We interfaced with a MIDI Unit (MOTU UltraLight Hybrid MK3) which recorded the two performances (with sound and without), plus it allowed the playback of the performance with sound during the listening-only condition. We positioned the piano keyboard on an adjustable desk at a comfortable height for the pianist. We positioned the eye-tracking computer’s screen on the same adjustable desk, behind and above the keyboard for optimal visibility. We attached the infrared camera of the ET unit to the lower edge of the computer screen, a flat DELL LCD monitor, with a screen resolution of 1,680 × 1,050. We presented the first two pages of each of the three pieces’ musical scores, after digitalizing them at a high-resolution, so that the pianist could read the score at a comfortable distance of about 50 cm. To facilitate playing and making the session more natural we did not use a chinrest, which is not problematic for the SMI eye-tracking equipment since it automatically corrects for changes in head position and rotation. Hence, in such testing conditions, it is possible to obtain reliable gaze data and mapped pupil diameter (in mm) that are free of artifacts due to head movements or changes in distance between the eyes and the screen.

A R.E.D. 250 SMI infrared eye and iView X Hi-Speed Software (SMI; Berlin, Germany) recorded eye positions at a sampling rate of 250 Hz. The RED can operate at a distance of 0.5–1.5 m. This device has two sources of infrared light from an infrared light-sensitive video camera, placed under the monitor frame. According to SMI, the RED system can detect changes as small as 0.004 mm. During the experiment, PP looked directly into the screen to the musical scores. We used BeGaze software from SMI to extract the gaze and pupil data, and Microsoft^®^ Excel, JASP, and Statview software for the statistical analyses.

#### Procedure

**Figure 1:**
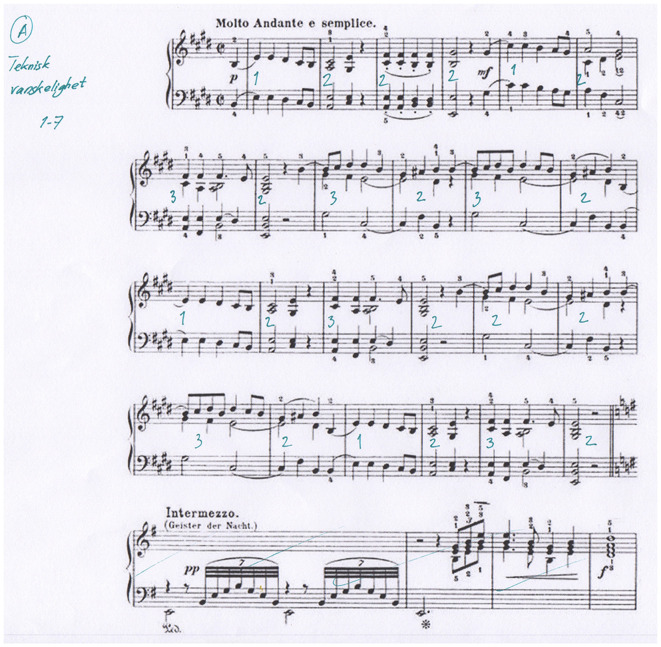
The score of Grieg’s Wächterlied for piano, with annotations by the professional pianist of its technical difficulty (measure by measure on a 7-step Likert scale).

A 4-point calibration procedure preceded each experimental session. The pianist always looked to the music score while playing as well as in the separate conditions, where PP imagined performing the same pieces “in her head.” Also, the pianist listened to her own playing of the same piano pieces (as recorded by MIDI from the keyboard and played back with headphones). One condition consisted of playing the piece while looking at the score on the screen with the muted electronic keyboard so that the hands/fingers’ movements produced no sound. At the end of all experimental sessions, the pianist rated levels of technical difficulty or expressivity and harmonic intensity on the musical score (by use of a 7-step Likert scale), measure-by-measure (see Figure 1 for an example).

To estimate subjective effort, at a later date after the pupillometry session, we asked the professional pianist to estimate with a 7-step rating scale each piece, measure by measure (roughly following the musical metric framework; see Keller, 2001), along with three different parameters of “difficulty”. One type we label “technical” (i.e., of the motor-related challenges of playing the notes as indicated in the score); then, the “expressive” (i.e., expressivity-related difficulties, like shaping the performance as intended); and finally, the “harmonic tension” (i.e., subjectively experienced harmonic tension and release).

### Results and Discussion

**Figure 2:**
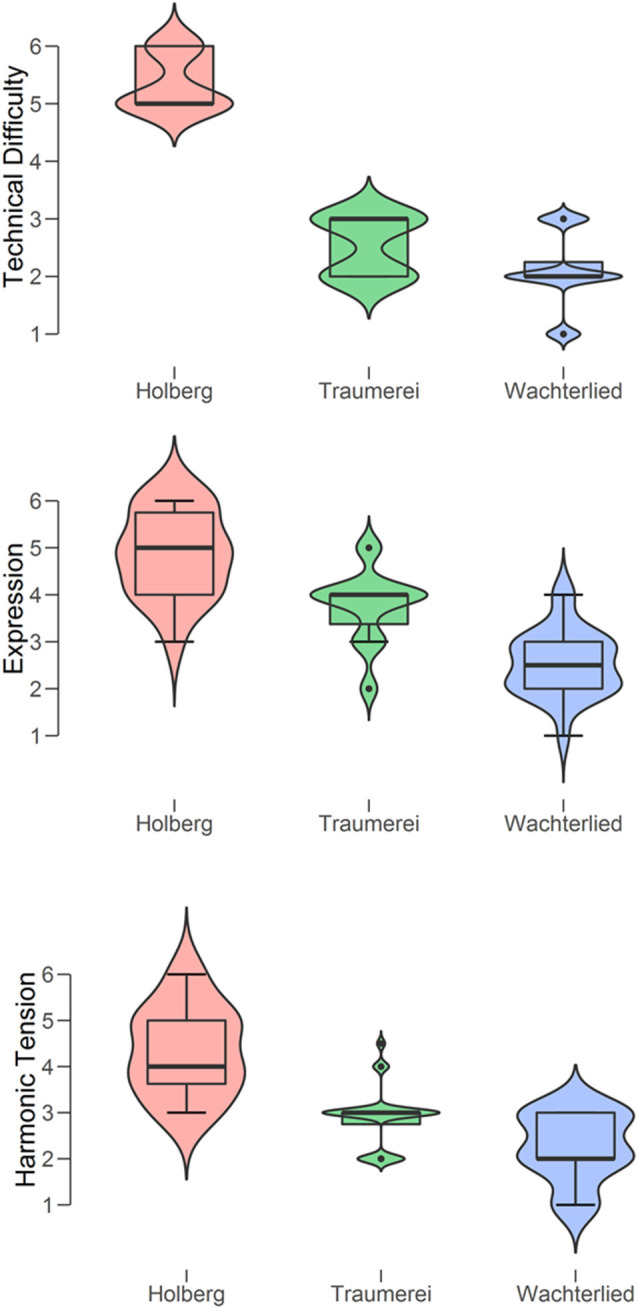
Violin boxplots of the ratings by the Professional Pianist of the three types of difficulty (from top to bottom: technical, expressive, and harmonic tension) for each of the three piano pieces (Holberg Suite, Träumerei, and Wächterlied).

We first computed descriptive statistics for the ratings given to each piece, measure by measure (see Figure 2). The pianist (PP) judged on Likert 7-step scales the technical difficulty, the expressivity, and the harmonic tension. Three separate ANOVAs with each of three ratings as dependent variables showed that all pieces differed from one another. Specifically: (a) technical difficulty, *F*_(2)_ = 194.1, *p* < 0.001, *η*^2^ = 0.86 (*post hoc* tests: 2.8 < *t* < 18.6; 0.02 < *p* < 0.001); (b) expression, *F*_(2)_ = 45.5, *p* < 0.001, *η*^2^ = 0.59 (*post hoc* tests: 4.5 < *t* < 9.5; all *p* < 0.001); (c) harmonic tension, *F*_(2)_ = 38.3, *p* < 0.001, *η*^2^ = 0.55 (*post hoc* tests: 2.9 < *t* < 8.7; 0.01 < *p* < 0.001). These mean ratings essentially confirm PP’s selection of three pieces in terms of pianistic challenges or performance demands, since consistently she rated the Holberg Suite highest on all three measures, Wächterlied was rated the lowest, while Träumerei was placed in between.

Interestingly, a multiple regression analysis with “technical difficulty” as the dependent variable and “expression” and “harmonic tension” as the independent variables revealed a strong positive relationship between the three measures, *F*_(2)_ = 85.5, *p* < 0.0001, *r* = 0.855. At the same time, these measures tap, as intended, on different aspects of the subjective effort in performance. Specifically, “technical difficulty” appeared to be more closely related to “harmonic tension” (Regression Coefficient = 0.865, *t* = 6.2, *p* < 0.0001) than to “expression” (Regression Coefficient = 0.280, *t* = 2.1, *p* = 0.04).

Our measure of objective (mental) effort was the pupil diameter during each condition. Since the pianist was looking at the same score in all conditions, we assume that the light stimulation to her eyes remained constant across musical measures and across conditions. In Figure 5 we show the waveforms of PP’s pupil diameter (as color lines) along time (in seconds) for each of the four conditions and for the three pieces (split in panels). These waveforms reveal several interesting aspects. First, the pupillary waveforms when “playing” (i.e., moving the fingers “with” or “without” sound) are consistently above the other two conditions where there is no movement (i.e., “listening” and “imagery”).

**Figure 3:**
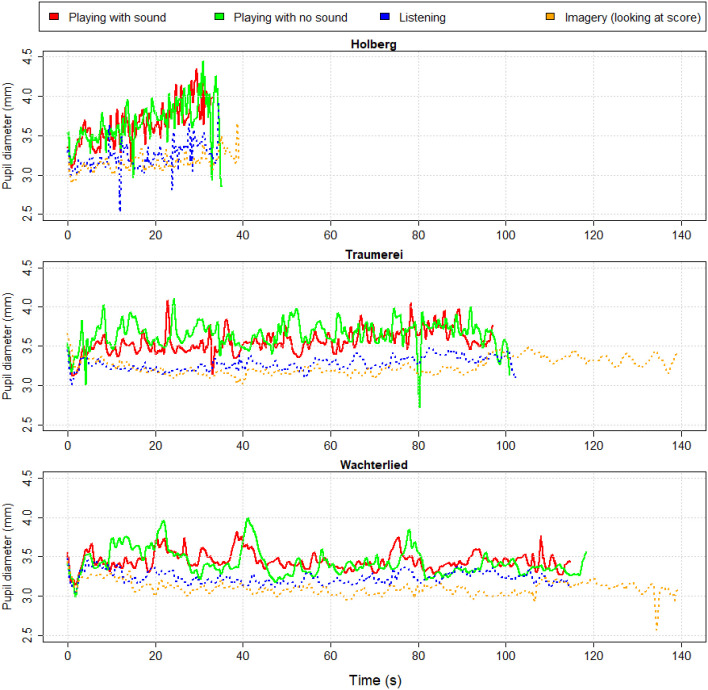
Pupillary waveforms of professional pianist (PP) during each of the three piano excerpts (Holberg Suite, Traäumerei and Wächterlied) and for each of the four conditions (“Playing with sound”—red line, or “Playing with no sound”—green line, “listening”—blue dotted line, and “imagery”—orange dotted line).

**Figure 4:**
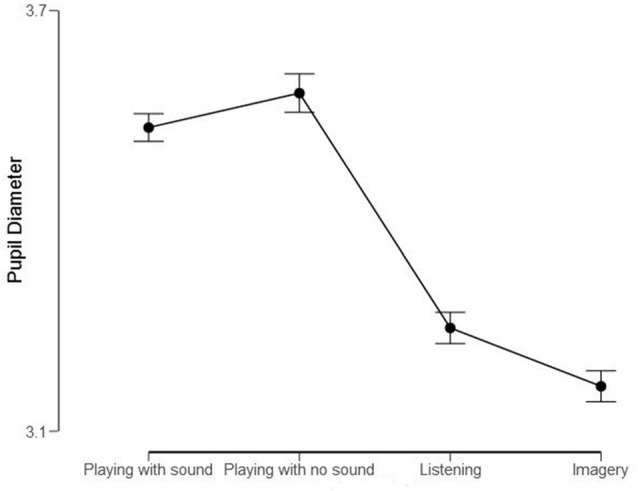
Mean pupil diameters (in mm) of the Professional Pianist for the four conditions. Bars represent 95% confidence intervals.

**Figure 5:**
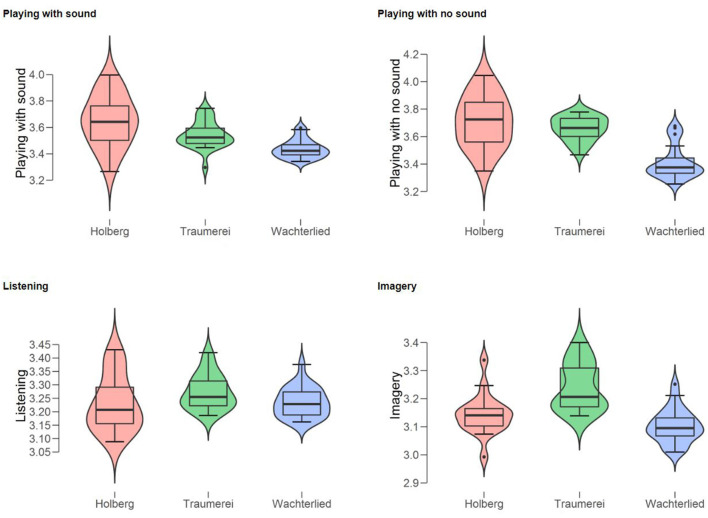
Violin boxplots of the mean pupil diameters (in mm) of the Professional Pianist for the three piano pieces (Holberg Suite, Träumerei, and Wächterlied) split by the four conditions (from top left panel: Playing with sound; Playing with no sound; Listening; and Imagery).

Second, it is clear that while the duration of each piece when playing either with or without sound differs only a few seconds from one another (and from listening), the imagery condition was—as seen in previous studies (e.g., Janata and Paroo, 2006)—longer than the other conditions. Traäumerei was imagined for about 40 s longer (i.e., a 40% lengthening) than when listening or performing. Similarly, Wächterlied was imagined for about 22 s longer (i.e., a 16% lengthening) than when listening or performing. PP performed the Holberg Suite at a faster rate than the other two pieces (in about 35 s) but, when imagined, its length stretched of about 5 s (i.e., a 14% lengthening).

We also computed descriptive statistics for mean pupil responses. Variations in pupil diameter approximate a normally distributed parameter (e.g., Mathôt et al., 2018). Moreover, *F*-tests remain robust also when data are not entirely normal (Blanca et al., 2017). Hence, we applied a repeated-measures ANOVA with the mean pupil diameter within each measure as the random factor and Conditions (Playing with sound; Playing with no sound; Listening; Imagery) as the within factor. This analysis revealed a significant effect of Conditions, *F*_(3)_ = 320.6, *p* < 0.001, *η*^2^ = 0.83. *Post hoc* (Bonferroni) tests confirmed that the four conditions differed significantly from one another (*p* < 0.001; Cohen’s *d* range: 0.4–2.68).

As visible in Figure 4, the most effortful condition—according to the pupil diameter—was “Playing with no sound,” followed by “Playing with sound”. The condition of “Listening” and “Imagery” were clearly less effortful (Cohen’s *d* > 2) compared to the previous “motoric” conditions. Looking at the score and imagining the music was the least demanding of all conditions.

Also, we run an ANOVA on the mean pupil diameters for the three musical pieces. This analysis revealed a significant effect of the factor of Musical Piece, *F*_(2)_ = 18.9, *p* < 0.001, *η*^2^ = 0.38. *Post hoc* (Bonferroni) tests showed that both the Holberg Suite (mean pupil diameter = 3.432; SD = 0.13) and Träumerei (mean pupil diameter = 3.429; SD = 0.07) differed significantly (*p* < 0.001) from Wächterlied (mean pupil diameter = 3.296; SD = 0.05). However, they did not differ from each other (see Figure 5 illustrating in boxplots the pupil diameter for each piano piece and each condition in separate panels).

A multiple regression analysis explored the relationship between the four conditions. One multiple regression used “imagery” as the dependent variable and the other three conditions as independent variables, which revealed a highly significant relationship and a moderate positive relationship, *F*_(3)_ = 17.5, *p* < 0.0001, *r* = 0.68. Specifically, “imagery” was highly significantly related to “listening” (Regression Coefficient = 0.65, *t* = 4.85, *p* < 0.0001). Imagery was also significantly related to “Playing with no sound” (Regression Coefficient = 0.224, *t* = 2.1, *p* = 0.04), but failed to reach significance with the standard performance condition or “Playing with sound” (Regression Coefficient = 0.12, *t* = 1.05, *p* = 0.30).

**Figure 6:**
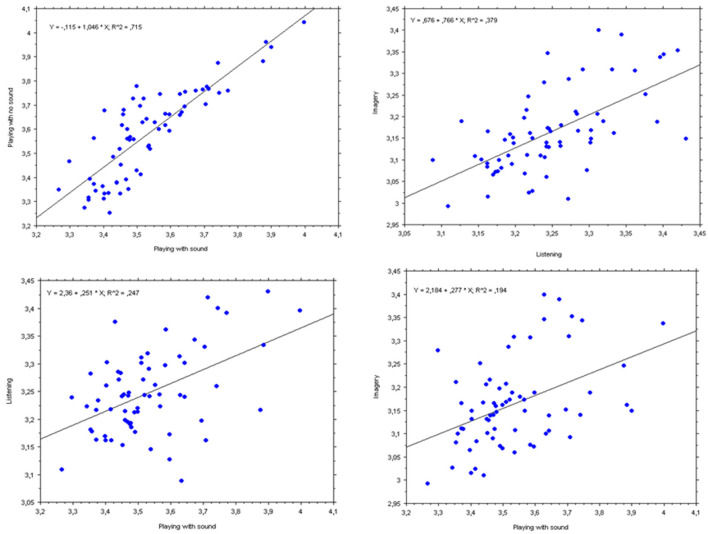
Bivariate plots and simple regression lines for PP’s mean pupil diameters (in mm) for each measure of all three pieces together. Top left panel: dependent variable = “Playing with no sound”; independent variable = “Playing with sound”; Top right panel: dependent variable = “Imagery”; independent variable = “Listening”; Bottom left panel: dependent variable = “Listening”; independent variable = “Playing with sound”; Bottom right panel: dependent variable = “Imagery”; independent variable = “Playing with sound”.

Figure 6 illustrates in detail how the mean pupil diameters (in each musical measure or bar) are related to each other in each condition. Specifically, we subdivided the pupil time series by the number of bars in the score, to standardize the pupil data between conditions differing in length. The results confirmed our expectation, based on the idea that each of the conditions would draw resources or demand mental effort, that the pupil changed similarly during the same moments (or “chunks,” i.e., measures) of a musical piece. Indeed, the two motoric conditions (Playing with sound and Playing with no sound), both requiring actual finger movements, showed the strongest relationship (Figure 6, top left panel), *F*_(1,64)_ = 160.6, *p* < 0.0001, *r* = 0.85. Most interestingly, the second strongest relationship was between “listening” and “imagery” (i.e., the two conditions without explicit motoric involvement), *F*_(1,64)_ = 39.1, *p* < 0.0001, *r* = 0.62. This positive relationship might be attributed to hearing the music, not only when listening, but also “in the mind”s ear’ when imagining.

The two next strongest relationships (displayed in the bottom panels of Figure 6) showed only moderate correlations, though both statistically significant. “Playing with sound” was positively related to “Listening” (bottom left panel), *F*_(1,64)_ = 20.9, *p* < 0.0001, *r* = 0.49. Remarkably, “Playing with no sound” (bottom right panel) was positively related to “Imagery,” *F*_(1,64)_ = 21.6, *p* < 0.0001, *r* = 0.50. All other simple regression had still positive correlations, but with Spearman’s coefficients *r* below 0.5, accounting for less than a quarter of the variance and are not displayed here.

In sum, our objective measure of effort, the pupil size, showed that pupil diameters were largest (Figure 4) when “playing” (regardless whether there was sound produced or not) and above the conditions where there is no movement (i.e., “listening” and “imagery”). This suggests that programming and executing motion is considerably more demanding than situations in which action is not required. This could also be partly because mental and physical efforts are meshed during the action and both could show up in the measure of attentive workload.

Although “musical effort” in general has been an understudied aspect of music cognition (see Keller, 2001), a study by O’Shea and Moran (2016, in Study II) examined explicitly how the eye pupil adjusted when pianists performed a piece but also while they simply imagined the performance. They reported no difference between their musicians’ pupils during the actual performance and its imagery, which seems consistent with an equivalent deployment of arousal for the same processes in the two conditions. However, they based such a conclusion on the results of an analysis of variance that barely missed the 0.5% cut-off (*p* = 0.053), which might not constitute conclusive evidence for no difference.

We confirmed that subjective effort is related but not identical to the objective effort, since the ordering of the two differed slightly (Figures 2, 5), especially for “listening” and “imagery.” Most interestingly, changes in the mental effort as the musical piece evolved (measure-by-measure) were all positively related (Figure 6) and their relationship was strong for the two playing conditions and moderate for the two non-playing conditions, revealing that regardless music is performed or only listened (with the physical ear or mind’s ear) the pupils’ diameter co-vary at each point in time. This can be interpreted as good evidence that all conditions share the same cognitive mechanisms and a similar workload along with the musical piece.

Finally, we replicated the finding that imagery can stretch the timeline. It is possible that when playing music in one’s head, there is the luxury to pause or dwell on a particular moment. Because imagery involves additional mechanisms of generation and maintenance as well executive processes (Glover et al., 2020), this could lengthen processing, without apparently increasing the cognitive workload compared to actually listening (Figure 4).

## Experiment 1B

In the following experiment, we sought to provide evidence that also listeners would react to the perceived “musical effort” inherent in the music and likely more in musicians than in non-musicians, while they listened to the piano renditions by our professional pianist. The spontaneous pupillary responses can be compared for similarity across groups and with those of the performer.

### Method

We used the same pupillometry method of Experiment 1A.

#### Participants

We recruited 20 participants (12 females) as volunteers for the listening group (mean age: 28.15 years, range: 19–65). Of these, 10 were pianists (mean age: 26.5 years, 7 females) and 10 were non-musicians (mean age: 29.8 years, 5 females), all based on self-reports. All participants read and signed informed consent.

#### Apparatus

We used the same R.E.D. 250 SMI infrared eye tracker in the same laboratory room used in Experiment 1A. However, we recorded eye positions at a sampling rate of 60 Hz, which is a sufficient sampling rate for pupil measurements (Laeng and Alnæs, 2019).

#### Procedure

A four-point calibration procedure preceded each experimental session. Participants looked at all times to a blank gray screen with a circle at the center (5 cm in diameter) while the music was playing as well as in a “baseline” recording, in silence right before the music, of 1 s. Participants seated in front of a monitor with their head supported on a chinrest and listened to recordings of the different pieces performed by PP (Holberg Suite, Träumerei, and Wächterlied). There were two different versions of each piece, one where PP listened to the sound produced by the piano (“Sound” or normal condition) and one where PP did not get to listen to the sound produced (“No sound” or silent condition). All participants received the following instruction: “Please look at the circle in the middle of the screen while listening to the music. Keep your eyes open during the experiment (you can blink as normal).”

### Results and Discussion

**Figure 7:**
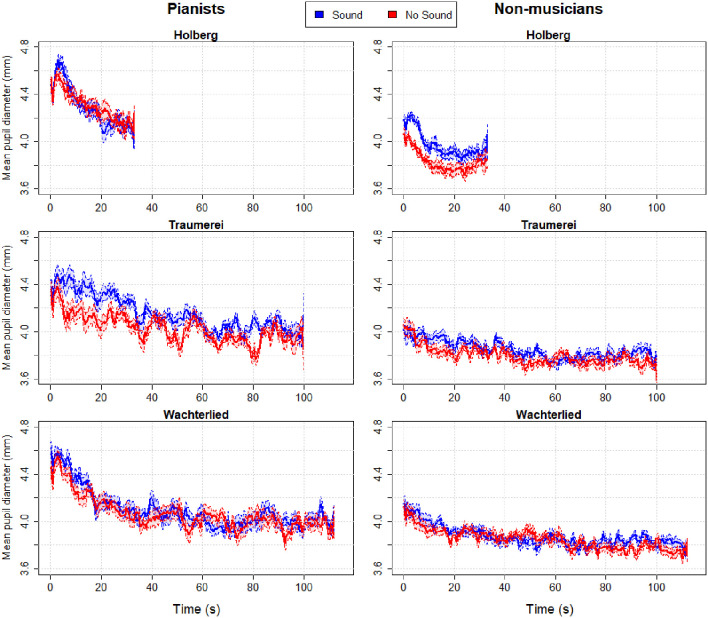
Pupillary waveforms of control participants after averaging the time series of pianists and non-musicians of the three piano excerpts. The two waveforms in each panel illustrate pupillary responses to listening to the two versions of each musical piece, one in which PP played with sound—i.e., standard playing condition (in blue)—and the other while listening to PP playing the same piece but with no sound or silent keys (in red). Patches along the curves indicate between-subjects (95%) confidence intervals.

Since half of the participants were not musicians, we collected only the measure of objective (mental) effort, i.e., pupil diameter during listening. In Figure 7 we show the waveforms of the Pianists’ and Non- musicians’ pupil diameters (split between the left side and right side panels respectively). The color lines show the pupil change along time (in seconds) for the two listening conditions and the three pieces (split in panels vertically).

Fidler and Loftus (2009; see also Loftus and Masson, 1994) have argued that graphs with (appropriate) error bars can replace significance tests. Hence, the plotted average pupil changes (in Figure 7) and their 95% confidence intervals reveal a tendency for larger pupil diameters when listening to the normal performance of PP, with sound, compared to her silent performance. This is particularly clear for the pianist group when listening to Träumerei and for the non-musicians group when listening to the Holberg Suite, which are the two most challenging piano pieces, whereas the easier Wächterlied shows no remarkable separation between the pupil waveforms. One interpretation is that the normal performance (where the pianist hears herself) is a more engaging rendition of the pieces than the silent one and therefore captures more attention from the listeners. A visual comparison of Figures 3, 7 (showing PP’s pupillary waveforms and, in particular, the blue lines of the “Listening” to the normal Playing) reveals that PP’s pupil maintained either a constant size (Traäumerei and Wächterlied) or increased over time (Holberg Suite). In contrast, both groups of control participants showed a reduction of their pupil diameters over time.

**Figure 8:**
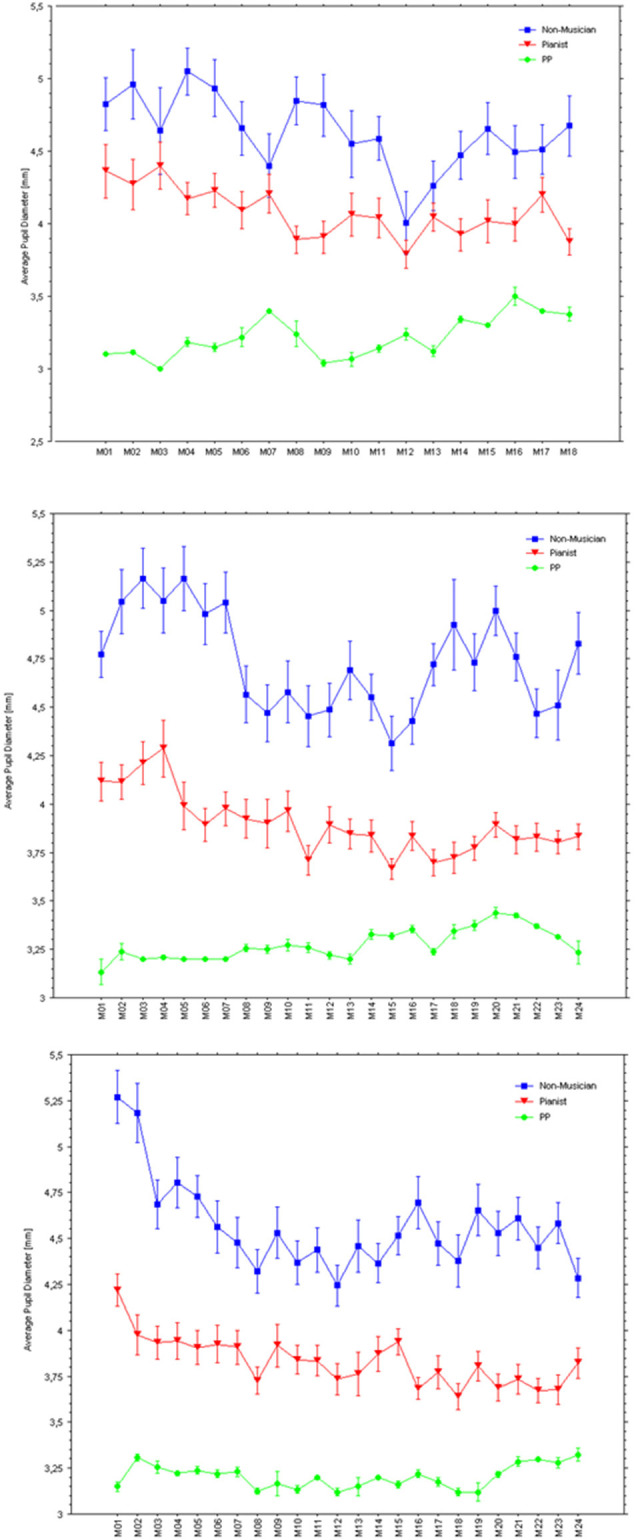
Average pupil diameters in mm (bars are SEs) within each measure while listening to PP’s playing with a sound each of the three pieces (Holberg Suite: top panel; Träumerei: middle panel; Wächterlied: bottom panel).

**Figure 9:**
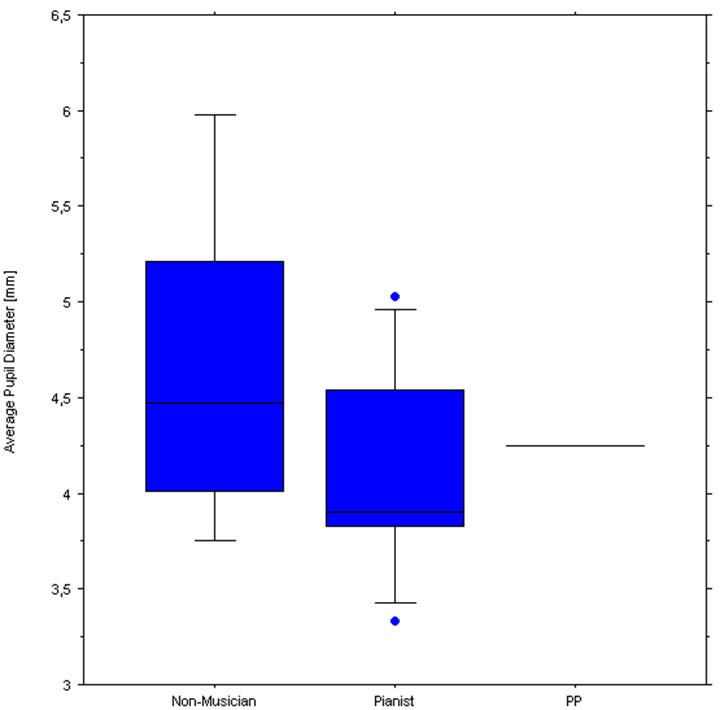
Boxplots of pupil diameters of the Non-musicians (left) and Pianists (middle) groups. The horizontal line to the right indicates PP’s baseline average pupil.

Figure 8 illustrates pupil responses during listening only to the normal playing of each piece, which reveals clear differences in pupil response between the two control groups and of both with respect to PP. Specifically, while the non-musicians’ average pupil was largest when listening to any of the three piano pieces, PP’s pupils showed the smallest responses. Figure 9 shows the baseline pupil measurements of the two control groups during a baseline pupil measurement at silent rest.

However, of special interest for the present investigation is to what extent the pupil responses, despite the strong difference in absolute response (probably reflecting how arousing was the music listening for the controls and the pianist), changed similarly with the unfolding of the music. Hence, we run multiple regression analyses using the change in average pupils as they occurred measure-by-measure, with PP’s average pupils as the dependent variable and Non-musicians’ and Pianists’ average pupils as the independent variables. The m-regression for the listening of the excerpt from the Holberg Suite showed no significant effects; *R* = 0.21, *F*_(2, 17)_ = 0.342, *p* = 0.72 (Regression Coefficients: Pianists = 0.16; non-musicians = 0.04). In contrast, the m-regression for the listening of Träumerei showed a significant effect, due to a significant relationship between pupil responses of the pianists and PP across measures; *R* = 0.52, *F*_(2, 23)_ = 3.82, *p* = 0.03 (Regression Coefficients: Pianists = 0.27; non-musicians = 0.01). The m-regression for the listening of the Wächterlied showed no significant effects; *R* = 0.30, *F*_(2, 23)_ = 1.05, *p* = 0.37 (Regression Coefficients: Pianists = 0.13; non-musicians = 0.10).

Also, we run separate multiple regression analyses using the change in average pupils as they occurred measure-by-measure, with PP’s and each of the control groups’ average pupils as the independent variables and as the dependent variable PP’s ratings (also measure-by-measure) in the three types of subjective effort) “technical difficulty,” “expression”, and “harmonic tension”).

The m-regressions for “technical difficulty,” during listening of the excerpt from the Holberg Suite, showed a significant effect, *F*_(3,17)_ = 3.74, *p* = 0.03. This was due to a positive relationship of subjective effort with the pupil responses of the Non-musicians (*p* = 0.02) but not with those of Pianists (*p* = 0.27) or PP (*p* = 0.95); *R* = 0.67 (Regression Coefficients: Non-musicians = 0.98; Pianists = 0.69; *PP* = 0.03). In contrast, the m-regression for “technical difficulty” and Träumerei showed no significant effects; *R* = 0.21, *F*_(3,23)_ = 0.31, *p* = 0.81 (Regression Coefficients: Non-musicians = 0.46; Pianists = 0.08; *PP* = 0.004). Similarly, there were no effects for Wächterlied; *R* = 0.29, *F*_(3,23)_ = 0.59, *p* = 0.63 (Regression Coefficients: Non-musicians = 0.92; Pianists = 0.62; *PP* = 0.008).

The m-regressions for “expression,” during listening of the excerpt from the Holberg Suite, showed no significant effects; *R* = 0.51, *F*_(3,17)_ = 1.6, *p* = 0.23 (Regression Coefficients: Non-musicians = 1.2; Pianists = 0.55; *PP* = 1.5). In contrast, the m-regression for “technical difficulty” and Träumerei showed significant effects for both Non-musicians (*p* = 0.009) and *PP* (*p* = 0.004); *R* = 0.70, *F*_(3,23)_ = 6.6, *p* = 0.003 (Regression Coefficients: Non-musicians = 1.75; Pianists = 0.02; *PP* = 5.9). However, there were no effects for Wächterlied; *R* = 0.41, *F*_(3,23)_ = 1.3, *p* = 0.29 (Regression Coefficients: Non-musicians = 0.16; Pianists = 1.6; *PP* = 3.4).

The m-regressions for “harmonic tension,” during listening of the excerpt from the Holberg Suite, showed no significant effects; *R* = 0.57, *F*_(3,17)_ = 2.2, *p* = 0.13 (Regression Coefficients: Non-musicians = 1.4; Pianists = 1.6; *PP* = 0.14). Similarly, the m-regression for Träumerei showed no significant effects; *R* = 0.30, *F*_(3,23)_ = 0.66, *p* = 0.59 (Regression Coefficients: Non-musicians = 0.37; Pianists = 0.78; *PP* = 2.6). Finally, there were no effects for Wächterlied; *R* = 0.49, *F*_(3,23)_ = 2.0, *p* = 0.13 (Regression Coefficients: Non-musicians = 0.89; Pianists = 0.55; *PP* = 2.69).

In sum, pianists listening to the performance would seem to attend more intensively to the most difficult piece than non-musicians, showing that their expertise with the same instrument could effectively engage their cognitive and perceptual system. Since pianists showed larger pupils than non-musicians only for the most difficult piece, it is unlikely that this difference was due to generally larger pupils within one group. Additionally, both the pianists and non-musicians seemed to be engaged more (Figure 7) by the standard performance (with sound) than the silent performance (which could have sounded less optimal).

Most interestingly, pupil sizes were smaller, when listening to all three pieces, for the professional pianist and largest for the non-musicians. Although this could be due to spurious pupil size differences across the groups and PP, we find this unlikely and we would like to suggest that these results might be indicative of expertise so that the amount of attention allocated for the same task is always lower in the expert or better performers than in novices.

Finally, there was weak evidence that, during listening, subjective effort measures (ratings) were related to the objective effort (pupil diameter), at least according to the timeline based on measures’ subdivisions. The only exceptions were the ability of the pupils of Non-musicians to predict “technical difficulty” for the Holberg Suite excerpt, and of the pupils of both Non-musicians and PP to predict “expression” for “Träumerei.”

## Experiment 2: fMRI

Several neuroimaging studies have specifically addressed music and imagery (e.g., Zatorre et al., 1996, 2007; Halpern and Zatorre, 1999; Satoh et al., 2001; Meister et al., 2004; Kraemer et al., 2005; Cebrian and Janata, 2010; Farrugia et al., 2015; Lu et al., 2017). In general, they reported activity within cortical structures, including the human motor or pre-motor cortex during imagery. For example, a study by Meister et al. (2004) showed that imagery and playing shared many sensory cortical areas, while bilateral primary motor areas were active only during playing. A few recent fMRI studies have specifically investigated piano players (e.g., Harris and de Jong, 2015), showing activations in the auditory and premotor cortex (PMC) during a motor imagery task compared to simply listening. Many studies converge in revealing that motor regions of the brain are involved in musical imagery and that imagery-like processes are involved in musical perception. However, to our knowledge, no studies have investigated the relationship between playing and imagining playing music when different levels of cognitive effort are involved and/or have specifically investigated the role of the subcortical region beside the cerebellum.

At least two single-case fMRI studies have previously investigated music listening, playing, and imaging in internationally renowned musicians, one with the popular artist Sting (Levitin and Grafton, 2016) and the other with the classical pianist Christopher Seed (Jäncke et al., 2006). The former confirmed substantial overlap of brain regions activated by listening and imagining, while the latter focused specifically on the ability of the classical pianist to play “left-handed” on a mirror keyboard compared “right-handed” on the standard keyboard.

Importantly, besides a study on jazz improvisation (Limb and Braun, 2008), no neuroimaging studies have explicitly examined mental effort in music-making. Most importantly, neuroimaging studies have not examined whether there is an activity within the LC in the brain’s brainstem while playing, listening, or imagining playing, that is in the area of the brain that has been directly related to mental effort in humans (Alnæs et al., 2014; Mäki-Marttunen et al., 2019). Because in the previous experiment, we applied the method of pupillometry as an objective measurement of mental effort during music listening, performance, and imagery, we have the opportunity to compare brain activity in the different conditions between PP and a control group, in the light of the results of the pupillometry experiment.

### Experiment 2A

We invited PP to participate in an fMRI experiment to assess the whole brain’s activity, including subcortical structures while listening, playing, and imagining piano pieces with a simple and complex level of cognitive and technical effort. Specifically, we used the same two Grieg’s pieces of Experiment 1, considered easy vs. difficult piano pieces, this also supported by PP’s subjective ratings (Figure 2) and—most importantly—by the pupil results (Figure 5) as shown earlier in Experiment 1. For practical reasons, we omitted during scanning the “playing without sound” condition, focusing on four conditions: listening, imagining the sounds, imagining playing, and actually “playing.” To enable a realistic “piano playing” condition, we set up a scanner-adapted piano keyboard, with sound played back through noise-canceling headphones. Our expectations derive from the literature that has revealed substantial overlap between cortical regions during imagery, listening, and playing, even at the single case level (Levitin and Grafton, 2016). Also, based on the results of Experiment 1, we expected to find activity in the brainstem’s LC that would differentiate the two levels of effort (easy vs. difficult). We also expected that brain activity would overlap more for listening and imagery than between these two and playing since PP’s pupil dilated the most during playing (Figure 4) and it was less active and in a similar way during the two other conditions (Figures 4, 6).

### Method

We used the initial part of the Wächterlied, from Lyrical Pieces, Op.12 No.3, and the Holberg Suite, Op. 40, Praeludium (Allegro vivace). In this and the following MRI experiments, adapted versions of subparts of the music sheets were presented on a computer screen, visible in the scanner, representing 15 s of the pieces. The pieces were also adapted to make it possible to play them on a small piano keyboard (with two octaves) in the scanner.

#### Participants

The target participant was the single case of PP who returned for testing with MRI about 1 year following the pupillometry experiment.

#### Apparatus

Scanning was performed with a Philips Achieva 3 Tesla MR scanner (Philips Medical Systems, Best, The Netherlands), equipped with an eight-channel Philips SENSE head coil. Functional data were collected using a BOLD-sensitive T2* weighted echo-planar imaging sequence [40 slices, no gap; repetition time (TR), 2.5 s; echo time (TE) = 30 ms; flip-angle = 80°; voxel size = 3 × 3 × 3; a field of view (FOV) = 240 × 240 mm; interleaved acquisition]. The slices were oriented to cover the whole cortex, cerebellum, and the brainstem’s pons. To avoid T1 saturation effects, five dummy scans were collected at the start of each fMRI run. Each run produced 340 volumes for each session. Anatomical T1-weighted images consisting of 184 sagittally-oriented slices were obtained using a turbo field echo pulse sequence (TR = 6.7 ms; TE = 3.1 ms; flip angle = 8°; voxel size = 1 × 1 × 1 mm; FOV = 256 × 256 mm). Also, to identify the LC, 39 transversally oriented slices of a high-resolution T1-weighted turbo spin-echo sequence were collected (TR = 600 ms; TE = 14 ms; flip angle = 90; voxel size = 0.4 × 0.5 × 3 mm; FOV = 220 × 178).

A polyphonic keyboard adapted and tested for a 3T MRI scanner (Jensen et al., 2017) was used for the experiments. It uses 25 full-size, keys covering two full octaves and it is designed ergonomically for the MRI scanner. The keyboard rests on the participant’s legs so that all the keys are reachable by moving the forearms within the MRI scanner. The keyboard was attached by a MIDI cable to Novation NIO 2/4 USB audio interface and then connected, *via* USB cable, to the Windows laptop, using Reaper software for generating audio. The output from Novation audio interface is in turn connected to the Eurorack UB 1002 Audio mixer. The windows computer runs the experiment on E-prime generating audio. The output audio from the E-prime computer is delivered into another input in UB 1002 mixer. To reduce the impact of the scanner’s noise, the audio mixer provides the ability to play the mono and stereo audio. The output from the Audio mixer is delivered through active noise-canceling headphones (OptoACTIVE).

#### Procedure

The experiment was designed after Experiment 1 but optimized for fMRI by making two separate sessions for the “imagining” vs. “listening” and “playing” vs. “listening” conditions. We adopted a block design structure with an equal length of 15 s for “Listening, Imagining (playing)” and “Playing.” These conditions were pseudorandomized, and a rest period of equal length introduced between the active blocks. The music sheets were presented using E-PRIME3 on a calibrated MRI compatible LCD screen (NNL LCD Monitor, Nordic Neurolab, Bergen, Norway) placed behind the scanner bore. In the “Listening and Imagining” session, PP was instructed either to listen to a music clip while viewing the music sheet or to imagine playing the same piece without moving fingers or hands. The “Listening and Playing” session was identical to the previous one, except that PP was instructed to play the music from the score sheet. In the “Imagining the sounds” condition, the participant was instructed to imagine hearing the melody in her head.

#### Analysis

Functional data were transferred to 4D nifti and motion-corrected using SPM 12 (Ashburner and Friston, 2005). For the whole-brain analysis, we normalized the anatomical images to the MNI template using the unified segmentation and normalization algorithm implemented in SPM12 (Ashburner and Friston, 2005). The resulting transformation parameters were then applied to the functional images. Images were smoothed with a Gaussian kernel of 8 mm FWHM. A general linear model/GLM) was estimated for each voxel with a canonical hemodynamic response function (HRF) for each condition of interest. The statistical parametric maps were thresholded at *P* values below *P* < 0.05 (FWE corrected) at cluster level for *t* = 5.27 for the cortical and *t* = 5.43 for the conjunction and clusters (extend threshold 55 voxels) derived from the fixed effects analysis.

For the brainstem analysis, the unsmoothed images were first analyzed in participant space using a general linear model (GLM) with six HRF-convolved regressors representing blocks of activation during the three conditions (Listening, Imagining Playing, and Playing) separated over the two musical pieces. To obtain precision in localizing LC we first resampled the high-resolution anatomical scan to the Spatially Unbiased Infratentorial Template (SUIT; Diedrichsen, 2007), covering the cerebellum and brainstem. Next, β-images resulting from the GLM were resampled into the same template space at a resolution of 2 × 2 × 2 mm. Finally, we applied a probabilistic mask of LC (Tona et al., 2017) to the unsmoothed *t*-map for all the conditions to identify significant voxels associated with LC. Correspondence between the LC mask and anatomical LC was ensured through visual inspection of the relevant region in the high-resolution anatomical scan. While all LC-specific analyses were performed on unsmoothed data, the SUIT-normalized contrast images were also smoothed with a 3D Gaussian kernel (4 mm FWHM) for illustration purposes.

### Results and Discussion

**Figure 10:**
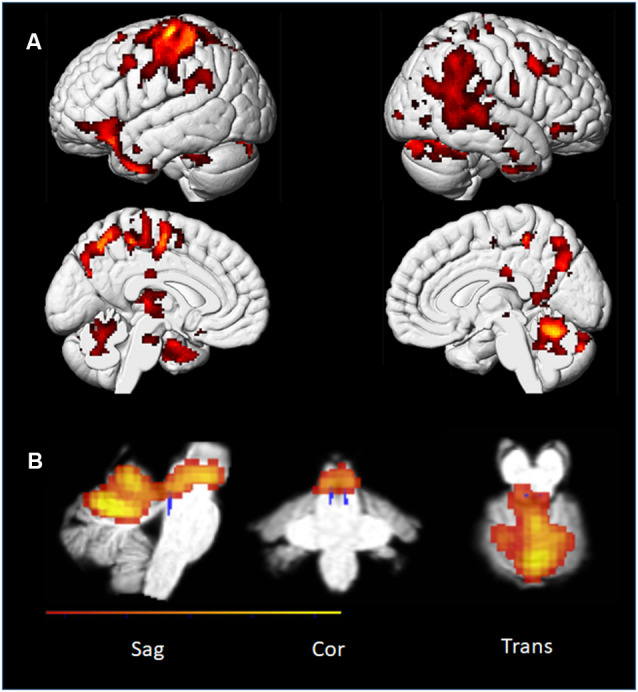
PP’s brain: *t*-maps representing areas showing activation when playing the difficult music piece contrasted to the simple. Maps are corrected for multiple comparisons using FWE (Cortical: *p* < 0.05, *k* = 55; and *p* < 0.05, *K* = 8 for the brainstem using the SUIT mask). **(A)** *t*-map projected on an MNI template cortical mesh. **(B)** Parametric map from the subcortical specific analysis (on a cropped version of a normalized image of PP). The locus coeruleus (LC) mask from Keren et al. (2009) is shown in blue. Brain stem activations are displayed on the SUIT template (Diedrichsen, 2007) using MRIcron (Rorden et al., 2007).

Our first aim was a whole-brain approach to imagery and effort. Specifically, we subtracted the activations related to the “simple” piano piece from the “complex” piano piece for all the conditions (listening, imagining playing, and playing; see Figure 10A). Thus, the T2*-weighted scans were not optimized for the identification of functional activity within such a tiny structure as the LC (see Turker et al., 2019 for a review on issues with identifying LC with fMRI). Still, by applying the LC mask and a small volume correction analysis approach, we found one significant voxel in the left and two in the right superior LC when comparing the two “playing” conditions (see Table 1). This fits with the visual inspection of the brainstem using the SUIT mask (Figure 10B). Even though these results must be regarded with caution, we suggest that our findings imply a role for LC related to cognitive load. For the other conditions, we did not find any significant voxels in this area. These results are consistent with the pupil findings that also showed clear differences in pupil size between the difficult Praeludium of the Holberg Suite, compared to Wächterlied and, in particular, during the “Playing” conditions (Figure 5).

**Table 1:** Brain areas activated for professional pianist (PP) in the three tasks.

		Location	BA	MNA Coordinates			kE
			*x*	*y*	*z*	
**Contrast of playing the complex piano piece compared to the simple piece**							
Left	Frontal	Postcentral	BA: 2, 3, 4, 5, 7	−28	−24	54	937
		Precuneus		−26	−24	66	909
		Supplementary Motor Area	BA: 6	−8	−14	50	143
		Superior Frontal		−26	−8	52	143
	Limbic	Uncus	BA: 20, 35	−36	14	−38	134
		Parahippocampal	BA: 38	−22	−2	−28	100
		Hippocampus	BA: 21	−30	4	−34	19
	Cerebellum	Cerebelum_Crus1, 2, Pyramis		12	−82	−30	38
	LC	Superior		−2	−36	−14	1*
Right	Temporal	Mid Temporal		42	−34	2	96
		Superior Temporal		48	−46	−4	38
		Inferior Temporal		50	−32	−2	12
	Limbic	Mid Cingulum	BA: 23	0	−22	28	79
	Cerebellum	Cerebelum_4_5_6		4	−64	−20	250
		Vermis_4, 5, 6, 8		8	−52	−12	283
	LC	Superior		4	−36	−14	2*
**Contrast imagining the complex compared to the simple piano piece**							
			NS		
**Contrast listening the complex compared to the simple piano piece**							
			NS		
**Conjunction of playing and imagining playing**							
Left	Frontal	Supplementary Motor Area	BA: 6	−4	−4	58	832
		Inferior Frontal Gyrus	BA: 44	−52	8	20	165
		Precentral Gyrus	BA: 6	−48	−6	48	240
Right	Occipital	Inferior occipital	BA: 18, 37	50	−66	−16	411
	Cerebellum	Crus 1		52	−68	−28	362
**Contrast of imagining compared to playing**							
			NS	
**Conjunction of imagining playing and listening**							
			NS	
**Contrast of imagining compared to listening**							
Left	Frontal	Supplementary Motor Area	BA: 6	−4	−4	58	744
		Frontal Superior	BA: 6	−26	−6	62	149
		Rolandic Operandis		−56	6	6	87
		Inferior Frontal Gyrus		−52	6	20	161
		Insula		−38	6	2	220
	Occipital	Fusiform Gyrus		−38	−76	−20	47
	Cerebellum	Crus_1, 6		52	−68	−28	368
Right	Frontal	Supplementary Motor Area	BA: 8	3	14	44	492
	Occipital	Middle Occiptal Gyrus	BA: 37	50	−66	−16	41
		Inferior Occipital	BA: 18	40	−80	−16	101

*The LC analysis was done as a small volume corrected analysis with peak activation at threshold of *F* = 0.001, *K* = 1.

Interestingly, we found no significant difference between the levels of difficulty for the Listening and Imagining Playing conditions in cortical activity, which underlines the fact that—at least at the single case level—monitoring brainstem’s activity may best relate to mental effort than the general cortical activity.

**Figure 11:**
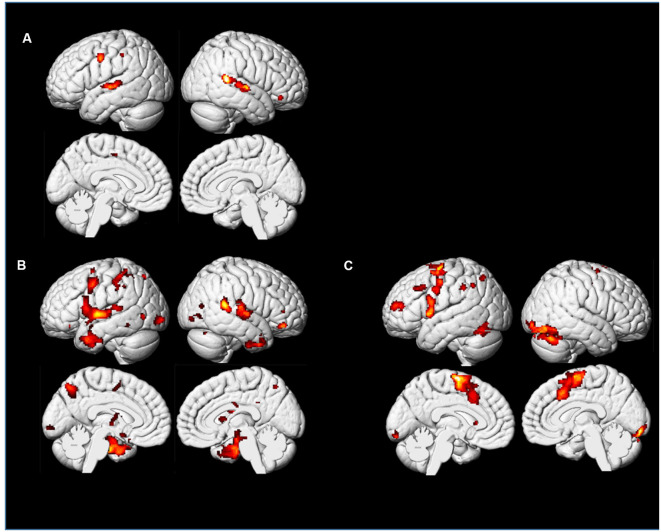
PP’s brain: *t*-maps representing PP’s cortical activations for Playing, Imagining Playing, and Imagining Listening. The maps were corrected for multiple comparisons using FWE (*p* < 0.05, *k* = 22). **(A)** Conjunction analysis of Imagining a melody and Listening to the same melody. **(B)** Conjunction analysis of Imagining Playing and actual Playing of the same piece. **(C)** The contrast between Imagining Playing a piece of music vs. Imagining Listening to it.

In the whole-brain analyses, the two levels of effort were analyzed together to increase statistical power. We found several cortical areas that were more strongly activated during the Playing condition compared to the others. Table 1 lists increased cortical activity in several areas associated with monitoring, motor planning, and motor execution. We found no significant result in the conjunction between Imagining Playing and Listening conditions suggesting that they imply different networks. Again, the pupil also differentiated Playing from Listening and Imagery (Figure 4) while there was a moderate relationship (Figure 6) between Listening and Imagery, suggesting some degree of overlap of the cognitive mechanisms and/or their degree of involvement in these conditions. We also found several areas where Imagining Playing provided more activations than the Listening condition. At the same time, we found that “Imagining” and “Listening” shared significant activations in the auditory cortex (see Figure 11A).

In summary, we did find consistently with the previous pupil findings that a complex piece of music contrasted to a simple piece lead to subcortical activations at the brain stem level. At the same time imagining playing shared cortical areas with actual playing (Figure 11 and Table 1; conjunction analyses) which was also consistent with several previous neuroimaging evidence on musical imagery (e.g., Zatorre et al., 2007; Zhang et al., 2017). Also, we confirmed that in our musician’s brain simply imagining a melody shared cortical areas with listening to the same melody.

## Experiment 2B

In the following MRI experiment, we looked at the brain activity in PP and a group of non-musicians when listening or imagining a well-known melody (“Happy Birthday”). In this MRI experiment, we necessarily excluded a condition with piano playing since that would not possible for such a non-musician group. In the “Imagine the melody” experiment, the participants listened to a simple well-known melody in blocks of 10 s and were asked to imagine listening to the same melody also in 10-s blocks. The conditions where pseudorandomized and a 10 s rest period were introduced between each block.

### Method

#### Participants

PP and eight control participants (five females; mean age = 26.2 years; range: 23–42). The control participants declared to be non-musicians; nevertheless, they all completed The Goldsmiths Musical Sophistication Index (v1.0), indicating an average score of 4.5 in musical training (it was 36 for PP) and a General Sophistication score = 48.8. The apparatus was the same as in Experiment 2A. The same prepossessing steps applied to the data. For the control participants, we derived the functional data based on the thresholded (*P* < 0.05, FWE corrected, *t* = 5.13) fixed effects data and extended the cluster threshold to 20 voxels.

#### Apparatus and Analysis

These were identical to the previous MRI experiment.

### Results and Discussion

**Figure 12:**
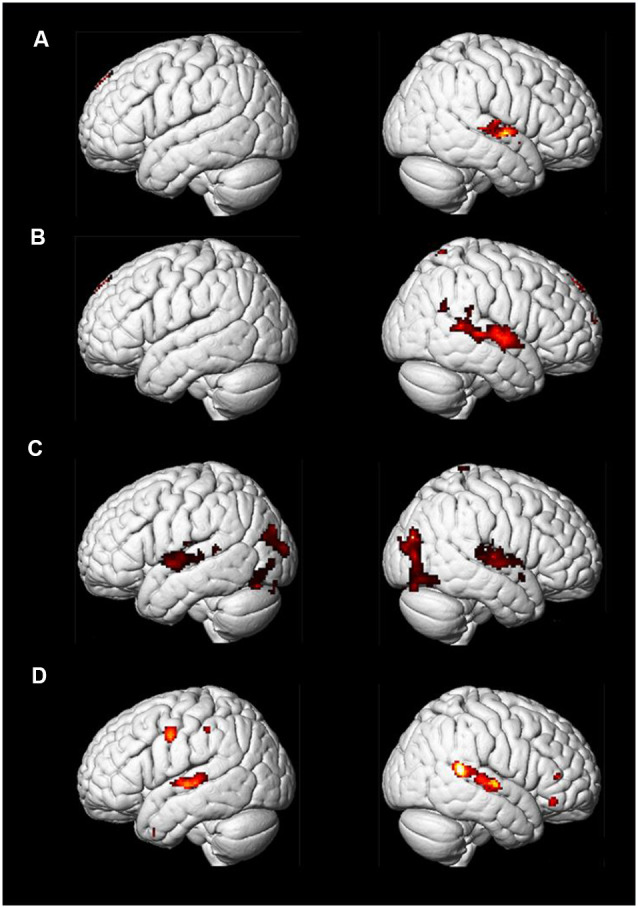
Control participants and PP: *t*-maps representing areas showing activations in the Imagining and Listening a well-known melody (“Happy Birthday”): Panel **(A)** shows the conjunction of Imagining and Listening for the group of non-musicians. Panel **(B)** shows the contrast between Imagining and Listening to the same melody. Panel **(C)** shows brain areas active when the non-musicians listen to a piano melody while looking at the musical score sheet showing the notes. Panel **(D)** show the same conditions as in Panel **(C)** but for PP only.

We found similar activations of “Imagining a melody” and “Listening to the melody” in PP’s auditory cortex, as confirmed by a conjunction model (Figures 11A, 12D). This is consistent, even at the single case level, with the widespread idea that imagery uses the same neural substrate of perception (Kosslyn, 1980, 1994; Zatorre et al., 1996; Halpern and Zatorre, 1999; Pearson et al., 2015). Interestingly, we found overlapping activations in the auditory cortex for non-musicians during the “Listening” condition, but we also found extended occipital activations (Figures 12A–C), within visual areas of the cortex, that were not revealed for PP. Although both PP and her controls were looking at the music score of the melody, there was likely less need of attending to this visual information for the professional pianist than for the non-musicians (see Schön et al., 2002). Moreover, as seen in Figures 11C, 12D, activations were in general lower for PP in the same areas than in the non-musicians. Lower neural activity in experts may seem paradoxical but it may be a hallmark of expertise (in musicians: Jäncke et al., 2000; Krings et al., 2000; Koeneke et al., 2004; but also in sport athletes: Naito and Hirose, 2014). That is, long-term training sharpens the relevant neural networks and dampens or filters irrelevant or noisy activity (Milton et al., 2007), so that the network becomes more efficient and uses lower activity or fewer dedicated units for its operation. Interestingly, the findings of Experiment 1B, where the pupil size when listening had an inverse relationship to expertise (Figure 8), seem consistent with the idea that experts require lower levels of effort, perhaps because the relevant neural network has become more efficient, than the less experienced or the novices.

## Discussion

The main goal of the present study was to examine “musical effort” or the cognitive workload that the act of imagining music, playing it, or listening to it, imposes on the mind or brain of a professional pianist as well as other individuals with different musical expertise. We used a multi-pronged approach, by use of both methods of pupillometry and magnetic resonance imaging, comparing these results between a professional pianist and groups of non-professional pianists or non-musicians. Although the present neuropsychological study is certainly limited, we believe that we succeeded in offering some initial but promising results about a few fundamental questions. First, we can measure musical effort through the eye pupil similarly to reading out cognitive workload and arousal in other domains. Second, musical imagery engages sensory and motor areas of the brain concerning the mental effort required by the complexity of the structure and execution of the imagined music. Third, musical imagery is similar to music listening in both non-musicians and musicians. Fourth, there is a degree of functional similarity between playing, imagining, and listening, when comparing both the activity in the NE system, as indexed by the pupil, and by the overlap in activity between sensory and motor neural networks in the whole brain. Finally, we revealed “audiation” in an expert musician with pupillometry, given the similarity in which the pupil changed during musical listening and imagery.

### Musical Effort as Revealed in the Eye Pupils

Experiment 1 provided clear evidence for a relationship between NE activity and mental workload or attentional intensity within the domain of music cognition. That is, the conditions requiring action were more effortful, regardless of the presence of auditory feedback than the conditions where no performance took place. Experiment 2 with fMRI confirmed in part this aspect since effort-related activations appeared most clearly in the playing condition with the professional pianist (PP) within the superior part of the LC. The fact that differences in the effort were not significant in the listening and imagery conditions of Experiment 2 can be explained by the fact that the latter two conditions engaged less the LC-NE system than the motor condition, as already shown by the small responses of the pupil in Experiment 1. The act of playing is also likely to add effortful motor planning and some degree of physical effort.

As expected, we found positive correlations between pupil diameters of the professional pianist during different “executions” of the same piano piece (i.e., normal, silenced, listened, and imagined), which also indicate similar task demands and differential degrees of load on resources. This might have also indicated the engagement of similar cognitive and execution mechanisms and, possibly, similar affective processes as the music unfolded. Our finding of a close affinity in cognitive workload between standard playing and silent playing confirms an intimate link between the motor imagery of sound-producing body motions and the resultant sounds (Godøy and Jørgensen, 2001).

Comparing a group of (non-professional) pianists and non-musicians while listening to PP’s performance of the three differently effortful pieces revealed several interesting aspects. Pianists attended more intensively to the most difficult piece than non-musicians since they showed larger pupils than non-musicians only for the most difficult piece. Non-musicians seemed to be the most engaged group by listening since their pupil size was larger overall than for the pianists as well as PP. This suggests that the amount of attention allocated for the same task may follow a hierarchy of expertise demanding less attentional effort in expert or performers than in novices.

Interestingly, there was only weak evidence for a commonality between subjective effort ratings and the objective effort gauged with pupil diameter during listening. The lack of a strong relationship suggests that psychophysiological methods like pupillometry index mental effort in a manner that is not “observable” in awareness or *via* introspection (Laeng et al., 2012; Laeng and Sulutvedt, 2014). Future studies should clarify to what extent subjective reports and objective measures of effort dissociate (e.g., Bruya, 2010). However, Kahneman (1973) had already pointed out that attentional effort is not identical to either “felt” effort or the observed likelihood of error. This is because effortless and overlearned tasks (e.g., telling someone one’s phone number) can visibly increase the pupil, revealing that the ease of retrieving information from long-term memory, instead of the load on working memory, lies behind the feeling of “effortlessness.”

### Musical Effort as Revealed by LC Activity

To our knowledge, the association between mental effort and LC activity in piano playing, albeit less in imagery, is the first reported in the literature. The brainstem region corresponding, according to current methods, to the anatomical coordinates of the LC were active when playing the “complex” piece by Grieg than the simpler piece, suggesting a role for NE activity varying with mental load (see Figure 10B). Caution must be taken in interpreting this result of course since this is a single subject study and there are several challenges involved both in identifying the LC areas and in interpreting fMRI results associated with LC. We, therefore, regard our findings as suggestive and promising for future studies to explore the connection between LC and mental effort.

Moreover, the fMRI results indicated that when PP imagined playing, this activated her brain’s premotor, motor, and perceptual areas, while when imagining the sounds of a melody (without playing) the auditive areas become predominantly active. This is consistent with previous studies of imagery: Bangert et al. (2006) found that a distributed network involving SMA and superior temporal gyrus activated during a muted keyboard task with pianists. Also, Gerardin et al. (2000) found evidence for overlapping neural networks responsible for imagined and real movements. Moreover, Bastepe-Graya et al. (2020) showed that imagery in an “oud” musician activated sensory and motor areas similarly when playing. Thus, the present study’s findings are also consistent with several previous neuroimaging studies (e.g., Langheim et al., 2002; Meister et al., 2004) by showing a substantial cortical overlap between playing and imagining music in a pianist and the presence of activity in the primary motor cortex during imagery. Importantly, these differential conjunctions in brain activity appear to mirror those exposed by pupillometry, where imagery appeared to be most similar to listening to the same piece in the professional pianist; a finding that we would like to interpret as capturing “audiation” on the fly.

## Limitations

The present findings should be considered cautiously since their generalizability may be limited given that we presented a single case, though of very high-level, pianistic, expertise. We also note that the control samples were small in both experiments, especially in the fMRI study. Hence, several of the present results are indicative rather than conclusive. Moreover, the study did not include a control experiment in which participants listened and imagined also non-musical contents, which could have thrown light on brain activity specifically related to music. The present study can be considered exploratory since it was motivated by a gap in music psychology research concerning mental effort and its role in musical performance and imagery.

## Conclusions

There is “musical effort” and it is measurable by the use of pupillometry. The use of pupillometry as a gauge of mental work in music is novel and the present findings suggest its potential. This objective method in measuring effort offers insights that may not be easy to expose by verbal reports or observing behavior. A combined and complementary approach of psychophysiology and neuroimaging seems very promising and it can provide converging evidence that considerably strengthens interpretations. In particular, activity in the brainstem’s LC modulated by task complexity is consistent with changes in the level of mental effort and NE neuromodulation as indexed by pupil size. Musical imagery has a strong commonality with music listening in both experts and naïve individuals.

## Data Availability Statement

The raw data supporting the conclusions of this article will be made available by the authors, without undue reservation.

## Ethics Statement

The study was reviewed and approved by the IRB of the Department of Psychology, University of Oslo (Ref. number: 3568281). The patients/participants provided their written informed consent to participate in this study. Written informed consent was obtained from the individual(s) for the publication of any potentially identifiable images or data included in this article.

## Author Contributions

BL, RG, and TE contributed to conception and design of the study. TE, MS, TH, AB, and BL performed the experiments and statistical analysis. TH and BL pre-processed the pupillometry data and TE pre-processed the fMRI data. BL wrote the first draft of the manuscript. TE wrote the fMRI section of the manuscript. All authors contributed to the article and approved the submitted version.

## Conflict of Interest

The authors declare that the research was conducted in the absence of any commercial or financial relationships that could be construed as a potential conflict of interest.
